# Characteristics of Chest HRCT and pulmonary function tests in elderly-onset primary Sjögren syndrome with interstitial lung disease

**DOI:** 10.1097/MD.0000000000032952

**Published:** 2023-02-22

**Authors:** Xin Dong, Yanli Gao, Man Li, Dong Wang, Jifeng Li, Yongfeng Zhang

**Affiliations:** a Department of Rheumatology, Beijing Chao-yang Hospital, Capital Medical University, Chaoyang District, Beijing, China; b Department of Radiology, Beijing Chao-yang Hospital, Capital Medical University, Beijing, China; c Department of Respiratory and Critical Care Medicine, Beijing Institute of Respiratory Medicine and Beijing Chao-Yang Hospital, Capital Medical University, Beijing, China.

**Keywords:** elderly-onset, interstitial lung disease, pulmonary function tests, radiography, Sjögren syndrome

## Abstract

To investigate the characteristics of elderly-onset primary Sjögren syndrome (pSS) using chest high-resolution computed tomography and pulmonary function tests (PFTs). The data of 102 patients with pSS with interstitial lung disease were retrospectively analyzed. The chest high-resolution computed tomography, PFTs, and clinical and laboratory data were evaluated based on the age of onset: elderly-onset pSS (EopSS) (≥65 years) versus adult-onset pSS (AopSS) (<65 years). Among the 102 patients with pSS-interstitial lung disease, there were 34 of EopSS and 68 of AopSS. EopSS patients presented a significantly higher incidence of usual interstitial pneumonia (EopSS [38.2%] vs AopSS [11.8%], *P* = .005) and a significantly lower incidence of nonspecific interstitial pneumonia (EopSS [8.8%] vs AopSS [25%], *P* = .042). Unlike the AopSS group, the significant decreases in the vital capacity (VC) (the percentage of the predicted value of each parameter [%pred]) and the forced VC (%pred), PFTs showed that VC (%pred) and forced VC (%pred) were >80% in the EopSS group. Forced expiratory volume in 1 second significantly decreased and residual volume significantly increased in the EopSS group (*P* = .001). The percentage of small airway disease was significantly higher in the EopSS group (*P* = .021). Diffusing capacity of the lung for carbon monoxide/alveolar volume (%pred) was <80% in both groups with a lower percentage in the AopSS group. Usual interstitial pneumonia is more common in the EopSS group. Although there is no significant difference in ventilation dysfunction between the EopSS and AopSS groups, small airway disease is more common in the EopSS group, while restrictive ventilatory dysfunction is more common in the AopSS group. Therefore, the EopSS group has its own characteristics and it is worth studying and noting.

**Key points**

- Aim: It is the first study to investigate the chest high-resolution computed tomography and pulmonary function test features in elderly-onset primary Sjögren syndrome (EopSS)-interstitial lung disease patients.
- Findings: Usual interstitial pneumonia is the most common pattern in the “lung-onset” subgroup of EopSS patients. Small airway disease is more common in the EopSS group.
- Message: The EopSS group has its own characteristics and it is worth studying and noting.

## 1. Introduction

Primary Sjögren syndrome (pSS) is a chronic progressive autoimmune disease characterized by lymphocytic infiltration of the exocrine glands, leading to significant loss of secretory function. The wide spectrum of pSS ranges from an organ-specific autoimmune disorder (autoimmune exocrinopathy) to a systemic process that involves musculoskeletal, pulmonary, gastrointestinal, hematologic, vascular, dermatologic, renal, and nervous systems.^[1]^ PSS can occur at all ages; however, it predominantly affects middle-aged women, typically in their fourth to sixth decades of life. An epidemiological study reported that the average age at diagnosis of pSS was 56.2 years with a female-to-male ratio of 9 to 1.^[2,3]^

Elderly-onset rheumatic diseases, such as rheumatoid arthritis (RA) and systemic lupus erythematosus (SLE), have distinct clinical and immunological characteristics.^[4,5]^ The incidence of elderly patients with autoimmune disease is increasing with increased life expectancies. Studies have indicated that age of onset can affect disease progression of autoimmune diseases, such as SLE and RA.^[6,7]^ Studies have shown that patients with elderly-onset pSS (EopSS) have lower levels of autoantibodies, such as rheumatoid factor (RF), antinuclear antibody (ANA), anti-Ro/SSA or anti-La/SSB antibodies.^[8,9]^

Elderly-onset pSS (EopSS) is associated with a higher incidence of interstitial lung disease (ILD).^[10]^ It is estimated that ~20% of pSS patients have an underlying ILD.^[11]^ Previous studies have reported that the average age of onset of pSS-ILD ranges from 58 to 68 years.^[12,13]^ Older age, lower forced vital capacity (FVC) and the usual interstitial pneumonia (UIP) pattern on high-resolution computed tomography (HRCT) can be used as prognostic factors for mortality in patients with pSS-ILD.^[12,14]^

We previously conducted clinical, laboratory, and lung imaging studies on 527 patients with pSS. We found that the mean age of pSS-ILD patients was (61.00 ± 11.23) years.^[15]^ To the best of our knowledge, no studies have been published on the ILD features of EopSS. The aim of this study was to characterize the ILD in EopSS by evaluating chest HRCT, pulmonary function tests (PFTs), clinical manifestations, and immune serological findings of patients with EopSS (age ≥ 65 years).

## 2. Materials and methods

### 2.1. Participants

**Figure 1.**
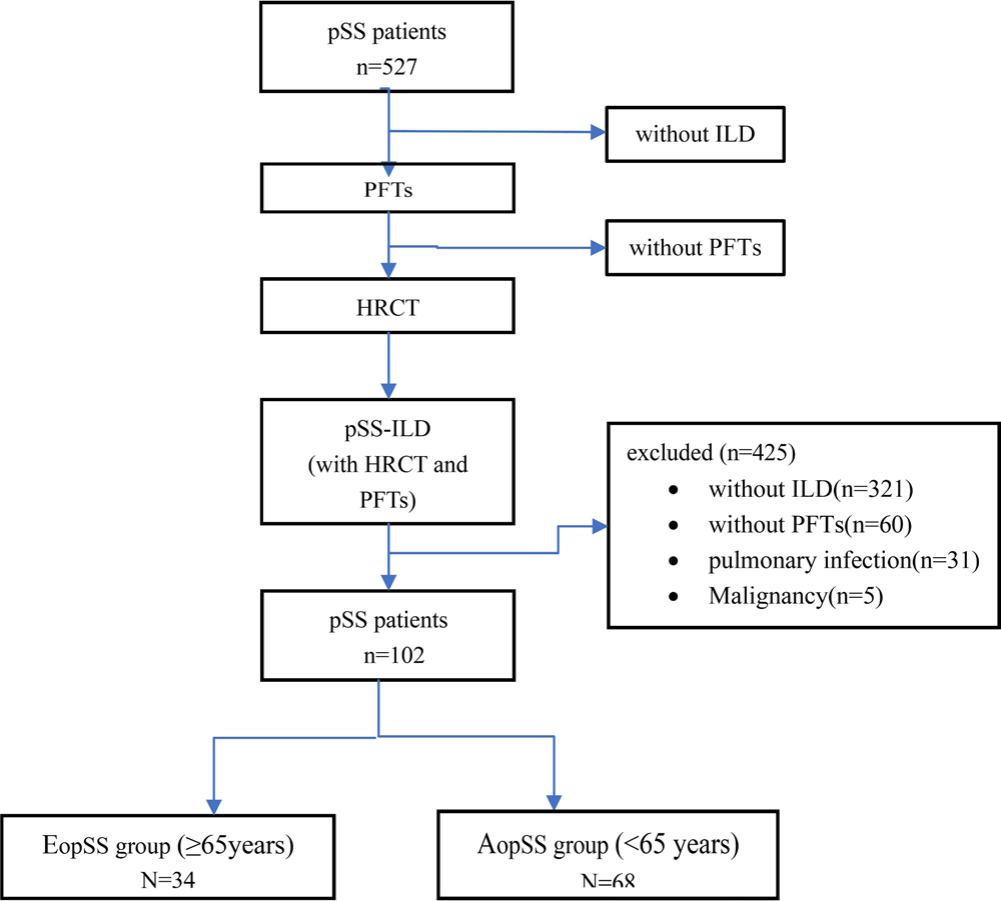
Flow chart of screening the study population. HRCT = high-resolution computed tomography, ILD = interstitial lung disease, PFTs = pulmonary function tests, pSS = primary Sjogren syndrome.

This was a retrospective study. From January 2011 to December 2020, in Beijing Chaoyang Hospital, Capital Medical University, 527 pSS patients were admitted, and 102 of them were eligible. Patients diagnosed with pSS based on the “2002 International Classification Criteria for Sjögren Syndrome”^[16]^ or “2016 American College of Rheumatology/European League against Rheumatism classification criteria for Sjögren syndrome,”^[17]^ were recruited. The diagnosis of pSS-ILD was based on abnormal HRCT for the presence of established ILD without infection, tumor, or occupational exposure. The pSS-ILD patients with the completion of both chest HRCT (Siemens, Germany) and PFTs (Master Screen Body, Germany) were included (Fig. 1).

The onset of pSS was defined as follows: initial clinical manifestation met the international classification criteria for Sjögren Syndrome (2 criteria); without oral and ocular symptoms, but having positive serum or supplementary examinations based on classification criteria. The onset of ILD was defined based on abnormal chest HRCT with the presence of established ILD with or without clinical symptoms.

### 2.2. Methods

#### 2.2.1. Clinical data and grouping.

The clinical and imaging data of the 102 patients with pSS-ILD included demographics (age at diagnosis and gender), disease history, history of diabetes and hypertension, history of solid tumors, history of smoking, clinical symptoms (oral symptoms, ocular symptoms, swelling of parotid gland, arthralgia, Raynaud phenomenon, fatigue, weight loss, cough, exertional dyspnea), physical signs (Rampant tooth, crackles), laboratory examinations, PFTs, and chest HRCT.

Laboratory examinations included the levels of immunoglobulins (IgG, IgM, and IgA), serum complement C (C3 and C4), C-reactive protein (CRP), RF, erythrocyte sedimentation rate (ESR), the presence of antibodies (ANA, anti-Ro/SSA, anti-La/SSB, etc), lactate dehydrogenase, albumin and tumor markers (cancer antigen 19-9 [CA19-9], cancer antigen 125 [CA125], carcinoembryonic antigen [CEA]).

pSS-ILD patients were divided into 2 groups according to the age of pSS onset: elderly-onset pSS (EopSS) (≥65 years) and adult-onset pSS (AopSS) (<65 years).^[8,10]^ The clinical and imaging data of these 2 groups were compared and analyzed. In the subgroup analysis stratified by initial symptoms, the 2 subgroups were named “sicca-onset” (oral and ocular symptoms) and “lung-onset” (the initial symptoms were pulmonary symptoms alone or in combination with oral and ocular symptoms), respectively.

#### 2.2.2. CT evaluation by radiologists.

CT images were obtained at full inspiration in the supine position for all patients and reconstructed by using a high-resolution reconstruction algorithm with contiguous 0.6-1mm sections. The classification of lung HRCT patterns was conducted by 2 radiologists with 20 and 10 years experience of chest CT diagnosis, respectively. HRCT patterns were categorized according to the 2013 international multidisciplinary classification of idiopathic interstitial pneumonia,^[18]^ as UIP, nonspecific interstitial pneumonia (NSIP), organizing pneumonia (OP), lymphocytic interstitial pneumonia (LIP) and “unclassified” subgroups. The radiological UIP criteria were applied as those for the diagnosis of idiopathic pulmonary fibrosis.^[19]^ NSIP was defined as the predominance of ground-glass opacity, possible visible subpleural sparing, and fine reticulation with minor or no honeycombing. OP was defined as single or multiple patchy consolidations. LIP was defined as multiple cysts and possible ground-glass opacity.

#### 2.2.3. Evaluation of lung functions.

The following PFTs data were analyzed: total lung capacity (TLC), vital capacity (VC), forced expiratory volume in 1 second (FEV1), FVC, residual volume (RV), forced expiratory flow (FEF25/50/75), and diffusing capacity of the lung for carbon monoxide/alveolar volume (DLCO/VA).

PFTs were defined as the percentage of the predicted value of each parameter (%Pred) for each individual based on age, gender, and height. The damages to lung functions mainly included obstructive ventilation disorder (FEV1/FVC [%] < 70% and RV/TLC [%] > 35%), restricted ventilation disorder (TLC [%Pred], VC [%Pred], and FVC [%Pred] < 80%), small airway obstruction (25% forced expiratory flow rate-50 [%Pred] < 70%, normal 75% forced expiratory flow rate [%Pred]), and diffusion dysfunction (DLCO/VA [%Pred] < 80%).^[20]^

### 2.3. Statistical analysis

SPSS 25.0 software (IBM SPSS Statistics 25) was used for statistical analysis. Quantitative data was expressed as mean ± standard deviation. Continuous variables were expressed as either mean ±  standard deviation or median and range. Independent samples *t* test or Mann–Whitney *U* test was used for 2 group comparisons. One-way analysis of variance (ANOVA) and least significant difference were used for multiple comparisons. Frequencies were analyzed using chi-squared test or Fisher exact test. Pearson correlation coefficient was used for the correlation between 2 continuous variables. Results were considered statistically significant if *P* < .05.

### 2.4. Ethical approval

Clinical and laboratory information was collected and approved by Beijing Chaoyang Hospital. Moreover, when the information was obtained, the patient’s work unit, home address, contact person, and telephone information were hidden, and there was no patient privacy exposure. This is a descriptive retrospective study. Therefore, this study has not been reviewed by the hospital ethics committee.

## 3. Results

### 3.1. Characteristics of participants

A total of 102 patients with pSS-ILD were included in the study cohort. Eleven patients were male (10.8%) and 91 patients were female (89.2%). The median age of ILD-onset was 62 (56, 67) years. The median age of the pSS-onset was 61 (54, 66) years. The duration of the disease was 0 to 40 years. Respiratory symptoms were prior to sicca in 10 cases. Thirty-four of the 102 pSS-ILD patients were assigned to the EopSS group (≥65 years) according to the age of pSS-onset, and 68 cases were assigned to the AopSS group (<65 years). The duration of the disease was 2 (0.31, 7) years for the EopSS group and 4 (0.35,7.75) years for the AopSS group, respectively. The patients who had respiratory symptoms were prior to sicca, 1 case in the EopSS group, and 9 cases in the AopSS group.

The percentage of males in the EopSS group was higher compared with the AopSS group, but there was no significant difference between these 2 groups (*P* > .05).

### 3.2. Clinical features of participants

Based on subgroup analysis stratified by initial symptoms, 34 cases (33.3%) were “sicca-onset” and 68 cases (66.7%) were “lung-onset.” In the EopSS group, 65% (22/34) patients were “lung-onset” and 35% (12/34) patients were “sicca-onset.” In the AopSS group, 67% (46/68) were “lung-onset” and 33% (22/68) were “sicca-onset.” There was no significant difference (*P* > .05) in the percentage of the “lung-onset” between the EopSS and AopSS groups (Table 3). AopSS group had a significantly higher frequency of arthralgia, compared with EopSS group (*P* = .042). There was no significant difference in Raynaud phenomenon, swelling of parotid gland, oral symptoms, ocular symptoms, fatigue, and weight loss between the AopSS and EopSS groups (Table 1).

**Table 1:** Clinical and laboratory variables in the elderly-onset and adult-onset primary Sjögren syndrome patients with interstitial lung disease.

	EopSS group	AopSS group	*P*
Clinical data			
N (%)	34 (33.3)	68 (66.7)	
pSS-onset age, yr	70 (66, 76)	55 (52, 61)	<.001
Respiratory symptoms-onset age, yr	71 (67, 77)	58 (53, 62)	<.001
ILD-onset age (median), yr	71 (66, 76)	56 (52, 61)	<.001
Course of disease (median), yr	2 (0.31, 7)	4 (0.35, 7.75)	.68
F/M	F:M (28:6)	F:M (63:5)	
Male, n (%)	6 (17.6)	5 (7.4%)	.109
N (%) (pSS duration ≤ 1 yr when ILD onset)	22 (64.7)	46 (67.6)	.466
Dry mouth, n (%)	29 (85.3)	48 (71.6)	.099
Dry eyes, n (%)	24 (70.6)	42 (62.7)	.510
Parotid enlargement, n (%)	0 (0)	5 (7.6)	.163
Rampant tooth	13 (38.2)	22 (32.4)	.555
Arthritis, n (%)	2 (5.9)	14 (20.9)	.042
Asthenia, n (%)	10 (29.4)	14 (20.9)	.458
Weight loss, n (%)	6 (17.6)	6 (9.0)	.212
Raynaud, n (%)	1 (2.9)	9 (13.4)	.089
Dry cough, n (%)	27 (79.4)	51 (76.1)	.805
Panting after activity, n (%)	24 (70.6)	48 (71.6)	.544
Crackles	24 (70.6)	43 (63.2)	.520
Laboratory data			
ANA ≥ 1:100, n (%)	30 (88)	57 (83.3)	.392
Anti-Ro/SSA52KD(+), n (%)	20 (58.8)	43 (63.2)	.412
Anti-Ro/SSA60KD(+), n (%)	13 (38.2)	31 (45.6)	.311
Anti-Ro/SSA(+), n (%)	23 (67.6)	53 (77.9)	.336
Anti-La/SSB(+), n (%)	7 (20.6)	13 (19.1)	.528
RF(+), n (%)	10 (29.4)	20 (16.7)	.577
Serum IgG, mg/dL	1780 (1365, 2277)	1560 (1280, 2070)	.069
High IgG, n (%)	23 (67.6)	35 (52.2)	.102
Serum IgA, mg/dL	353.5 (239.8, 504.5)	305 (215, 383)	.239
High IgA, n (%)	10 (29.4)	10 (14.9)	.074
C3, mg/dL	94.7 (81.4, 94.7)	99 (89.2, 110)	.547
Low C3, n (%)	8 (23.5)	7 (10.6)	.080
C4, mg/dL	18.6 ± 6.3	21.3 ± 6.5	.046
Low C4, n (%)	4 (11.8)	3 (4.5)	.176
ESR, mm/h	40.5 ± 27.7	26.8 ± 19.4	.005
High ESR, n (%)	27 (79.4)	49 (72)	.290
CRP, mg/dL	0.66 (0.29, 2.2)	0.48 (0.3, 1.0)	.191
High CRP, n (%)	15 (44.1)	23 (33.8)	.212
CA19-9, U/mL	14.1 (7.65, 28.5)	7.42 (4.4, 18.0)	.035
CA125, U/mL	16.9 (12.5, 33.8)	17.0 (11.1, 28.9)	.792
CEA, ng/mL	2.37 (1.47, 3.53)	1.77 (1.03, 2.65)	.207
LDH, U/mL	224.12 ± 77.5	208.8 ± 70.5	.320
ALB, g/L	34.00 ± 5.4	35.31 ± 4.2	.181

Data are presented as either mean ± standard deviation or median (Q1, Q3) unless otherwise stated.

ALB = albumin, ANA = antinuclear antibody, AopSS = adult-onset primary Sjögren syndrome, C3 = complement 3, CA125 = cancer antigen 125, CA19-9 = cancer antigen 19-9, CEA = carcinoembryonic antigen, CRP = C-reactive protein, EopSS = elderly-onset primary Sjögren syndrome, ESR = erythrocyte sedimentation rate, F/M = female to male ratio, N (%) = the number of cases (percentage), High IgG, n (%) = the ratio of higher than normal value, IgG = immunoglobulins G, IgA = immunoglobulin A, ILD = interstitial lung disease, LDH = lactate dehydrogenase, Low C3, n (%) = the ratio of lower than normal value, RF = rheumatoid factor.

**Table 3:** The patterns of chest high-resolution computed tomography in the “lung onset” and “sicca onset” groups.

	EopSS group		AopSS group			
	UIP	Non-UIP	UIP	Non-UIP	Total	*P*
Lung onset n (%)	9 (40.9)	13 (59.1)	4 (8.7)	42 (91.3)	68 (66.7%)	.003
Sicca onset n (%)	4 (33.3)	8 (66.7)	4 (18.2)	18 (81.8)	34 (33.3%)	.279
Total n (%)	13 (38.2)	21 (61.8)	8 (11.8)	60 (88.2)	102 (100%)	.005

AopSS = adult-onset primary Sjögren syndrome, EopSS = elderly-onset primary Sjögren syndrome, UIP = usual interstitial pneumonia.

### 3.3. Laboratory data

ESR was significantly higher and C4 was significantly lower in the EopSS group, compared with the AopSS group (*P* < .05). Correlation analysis showed that the age of onset was positively correlated with ESR (*R* = 0.257, *P* = .009) and CRP (*R* = 0.206, *P* = .038), respectively (Table 1). EopSS group had a significantly higher level of serum CA19-9, compared with the AopSS group (*P* = .035). There was no significant difference in the CA125 and CEA. Correlation analysis showed that age was positively correlated with the CA19-9 level (*R* = 0.203, *P* = .05) (Table 1).

### 3.4. Comparisons of ILD and PFTs according to the onset-age of pSS

The HRCT data from 102 patients with pSS-ILD were shown in Table 2. The most common HRCT finding was that multiple HRCT patterns coexisted with OP, NSIP, and UIP, followed by the single HRCT pattern, such as UIP, NSIP, unclassified pattern, OP, and LIP. In all pSS-ILD patients, the main HRCT patterns of NSIP or OP were 52 (51%) cases and 37 (36%) cases, respectively. In the EopSS group, the most common HRCT finding was UIP, followed by multiple patterns, unclassified patterns, NSIP/OP, and LIP. In the AopSS group, the most common HRCT finding was the multiple HRCT patterns, followed by NSIP, UIP, unclassified pattern, and OP/LIP (Table 2). The percentage of UIP was higher in the EopSS group (*P* = .005). The percentage of NSIP and Multiple with NSIP features were higher in the AopSS group (*P* = .042, *P* = .035) (Table 2). Based on the subgroup analysis, the prevalence of UIP in the EopSS group with “lung-onset” was significantly higher, compared with the AopSS group (*P* = .003) (Table 3).

**Table 2:** The patterns of chest high-resolution computed tomography and the index of pulmonary function tests in the elderly-onset and adult-onset primary Sjögren syndrome patients with interstitial lung disease.

	Total	EopSS group	AopSS group	*P*
HRCT pattern, n (%)	102	34 (33.3)	68 (66.7)	.024
UIP (single)	21	13 (38.2)	8 (11.8)	.005
NSIP (single)	20	3 (8.8)	17 (25.0)	.042
OP (single)	8	3 (8.8)	5 (7.5)	.795
LIP (single)	3	0	3 (14.7)	.214
Multiple (OP/NSIP/UIP)	35	9 (26.5)	26 (38.2)	.238
With NSIP feature	32	9 (26.5)	23 (33.8)	.451
With OP feature	29	8 (23.5)	21 (30.9)	.438
Unclassified	16	6 (17.6)	10 (14.7)	.700
Custom grouping				
NSIP (single) + multiple with NSIP	52	12 (35.2)	40 (58.8)	.035
OP (single) + multiple with OP	37	11 (32.4)	26 (38.2)	.664
PFT index				
TLC, (%Pred)	78.61 ± 18.69	82.04 ± 16.53	76.84 ± 19.59	.189
VC, (%Pred)	85.45 ± 23.07	95.4 ± 24.7	80.3 ± 20.5	.002
FVC, (%Pred)	86.21 ± 24.19	96.6 ± 26.7	81.0 ± 21.2	.002
FEV1, (%Pred)	82.34 ± 21.97	90.3 ± 24.3	78.3 ± 19.6	.009
FEV1/FVC (%)	80.50 ± 8.29	76.60 ± 8.48	82.48 ± 7.51	.001
RV/TLC (%)	41.66 ± 8.14	45.49 ± 6.97	39.75 ± 8.05	.001
MEF25, (%Pred)	61.48 ± 29.38	63.8 ± 33.5	60.3 ± 27.3	.614
MEF50, (%Pred)	67.34 ± 27.37	60.2 ± 26	70.9 ± 27.5	.065
MEF75, (%Pred)	83.69 ± 35.11	72.5 ± 36.4	89.2 ± 33.4	.024
DLCO/VA, (%Pred)	68.08 ± 24.95	72.6 ± 25	65.8 ± 24.8	.193
RVD, n (%)	41	12 (35.3)	29 (42.6)	.526
OVD, n (%)	18	4 (11.8)	14 (20.6)	.409
SAO, n (%)	49	22 (64.7)	27 (39.7)	.021

Data are presented as either mean ±  standard deviation or median (Q1, Q3) unless otherwise stated.

(%Pred) = the percentage of the predicted value of each parameter, AopSS = adult-onset primary Sjögren syndrome, EopSS = elderly-onset primary Sjögren syndrome, DLCO/VA = diffusing capacity of the lung for carbon monoxide/alveolar volume, FEV1 = forced expiratory volume in 1 s, FVC = forced vital capacity, HRCT = high-resolution computed tomography, LIP = lymphocytic interstitial pneumonia, MEF25 = 25% forced expiratory flow rate, MEF50 = 50% forced expiratory flow rate, MEF75 = 75% forced expiratory flow rate, NSIP = nonspecific interstitial pneumonia, OP = organizing pneumonia, OVD = obstructive ventilatory dysfunction, PFT = pulmonary function test, RVD = restricted ventilation disorder, SAO = small airway obstruction, TLC = total lung capacity, UIP = usual interstitial pneumonia, VC = vital capacity.

All pSS-ILD patients completed the PFTs. One-way ANOVA showed that PFTs indexes (TLC [%Pred], VC [%Pred], FVC [%Pred], FEV1 [%Pred], FEV1/FVC [%], and RV/TLC [%]) were statistically significant (*P* < .05). TLC (%Pred), VC (%Pred), FVC (%Pred) and FEV1 (%Pred) were significantly higher in the pSS-LIP group (*P* < .05) (Table 4). One-way ANOVA followed by an least significant difference test was used to compare PFT indexes among different HRCT patterns (Table 4). TLC (%Pred), VC (%Pred), and FVC (%Pred) were <80% in the OP and multiple groups (*P* < .05). RV/TLC (%) was >35% in all patterns. RV/TLC (%) was significantly >40% and FEV1/FVC (%) was <80% in UIP and LIP. Compared with NSIP, 75% forced expiratory flow rate (%Pred) significantly decreased in the OP and multiple patterns. DLCO/VA (%Pred) was <80% in all patterns (*P* > .05).

**Table 4:** The index of pulmonary function tests in the different patterns of chest high-resolution computed tomography in the primary Sjögren syndrome with interstitial lung disease.

	UIP	NSIP	OP	LIP	Multiple	Unclassified	*P*
TLC, (%Pred)	80.66 ± 12.93	90.98 ± 17.43*	67.70 ± 17.67†	102.03 ± 7.78*‡	69.28 ± 16.29*†§	85.02 ± 20.16‡	<.001
VC, (%Pred)	91.16 ± 17.51	98.89 ± 26.30	76.14 ± 26.00†	106.57 ± 5.36‡	75.56 ± 19.24*†§	86.05 ± 24.37	.003
FVC, (%Pred)	92.86 ± 18.92	99.07 ± 29.76	77.00 ± 27.30†	107.63 ± 0.80†‡	76.60 ± 20.30*†§	86.61 ± 22.84	.005
FEV1, (%Pred)	88.87 ± 17.68	92.34 ± 26.59	72.59 ± 27.26†	99.90 ± 5.50‡	75.53 ± 18.60*†	79.00 ± 21.28	.021
FEV1/FVC (%)	77.49 ± 6.01	80.08 ± 7.73	78.91 ± 5.98	78.10 ± 4.35	84.51 ± 7.98*	77.40 ± 10.77	.014
RV/TLC (%)	42.50 ± 6.89	37.38 ± 10.15	38.31 ± 7.88	43.43 ± 6.32	41.95 ± 7.49	46.07 ± 7.29	.046
MEF25 (%Pred)	63.77 ± 42.15	50.96 ± 19.45	53.04 ± 21.36	67.40 ± 23.20	63.73 ± 23.79	68.01 ± 34.42	.547
MEF50 (%Pred)	72.64 ± 28.60	68.15 ± 19.93	49.49 ± 13.56	77.10 ± 34.35*	68.86 ± 28.79	63.62 ± 32.66	.428
MEF75 (%Pred)	86.60 ± 35.70	103.54 ± 23.72	70.20 ± 37.61†	93.30 ± 55.68	79.45 ± 31.92†	73.09 ± 41.53	.107
DLCO/VA, (%Pred)	70.75 ± 28.86	65.71 ± 25.12	69.20 ± 27.93	78.93 ± 14.77	67.92 ± 23.96	65.03 ± 24.10	.946

Data are presented as either mean ± standard deviation unless otherwise stated.

(%Pred) = the percentage of the predicted value of each parameter, DLCO/VA = diffusing capacity of the lung for carbon monoxide/alveolar volume, FEV1 = forced expiratory volume in 1 s, FVC = forced vital capacity, LIP = lymphocytic interstitial pneumonia, MEF25 = 25% forced expiratory flow rate, MEF50 = 50% forced expiratory flow rate, MEF75 = 75% forced expiratory flow rate, NSIP = nonspecific interstitial pneumonia, OP = organizing pneumonia, RV = residual air volume, TLC = total lung capacity, UIP = usual interstitial pneumonia, VC = vital capacity.

* compare with UIP, *P* < .05; † compare with NSIP, *P* < .05; ‡ compare with OP, *P* < .05; §, compare with LIP, *P* < .05.

PFT indexes were compared according to the age of the pSS-onset (Table 2). TLC (%pred), VC (%pred), and FVC (%pred) were all >80% in the EopSS group. VC (%pred) and FVC (%pred) significantly decreased in the AopSS group, compared with the EopSS group (*P* < .05). FEV1/FVC (%) significantly decreased and RV/TLC (%) significantly increased in the EopSS group, compared with the AopSS group (*P* < .05).The percentage of small airway disease was significantly higher in the EopSS group (EopSS [64.7%] vs AopSS [39.7%], *P* = .021). DLCO/VA (%pred) was <80% in both groups, and there was no significant difference between these 2 groups.

There were 68 cases with “lung-onset.” Analyses of PFTs indexes were conducted based on the initial symptoms with “lung-onset” (Table 5). TLC (%pred), VC (%pred), FVC (%pred), and FEV1 (%Pred) were >80% in the EopSS group, which was significantly higher, compared with the AopSS group (*P* < .05). The 25% forced expiratory flow rate (%Pred) and 50% forced expiratory flow rate (%Pred) were <70%, while DLCO/VA (%pred) was <80% in both groups. There was no difference between these 2 groups.

**Table 5:** The index of pulmonary function tests in the primary Sjögren syndrome with interstitial lung disease with the “lung onset.”

	EopSS group	AopSS group	*P*
N	22	46	
TLC, (%Pred)	82.95 ± 16.03	73.59 ± 18.72	.049
VC, (%Pred)	95.47 ± 19.25	77.57 ± 18.20	<.001
FVC, (%Pred)	95.59 ± 19.60	78.26 ± 19.23	.001
FEV1, (%Pred)	89.26 ± 15.85	75.55 ± 17.05	.002
FEV1/FVC (%)	75.87 ± 7.95	82.20 ± 6.58	.001
RV/TLC (%)	44.15 ± 7.84	39.62 ± 8.75	.048
MEF25, (%Pred)	65.56 ± 37.76	59.14 ± 26.29	.429
MEF50, (%Pred)	62.60 ± 28.80	67.37 ± 23.63	.481
MEF75, (%Pred)	74.52 ± 39.10	87.12 ± 32.64	.177
DLCO/VA, (%Pred)	69.39 ± 24.39	64.65 ± 26.57	.484

Data are presented as either mean ± standard deviation unless otherwise stated.

(%Pred) = the percentage of the predicted value of each parameter, AopSS = adult-onset primary Sjögren syndrome, EopSS = elderly-onset primary Sjögren syndrome, DLCO/VA = diffusing capacity of the lung for carbon monoxide/alveolar volume, FEV1 = forced expiratory volume in 1 s, FVC = forced vital capacity, MEF25 = 25% forced expiratory flow rate, MEF50 =50% forced expiratory flow rate, MEF75 = 75% forced expiratory flow rate, RV = residual air volume, TLC = total lung capacity, VC = vital capacity.

## 4. Discussion

The number of elderly patients with autoimmune rheumatic disease is increasing due to increase in life expectancy and advanced medical technology. The age of onset of autoimmune diseases can impact the prognosis and clinical features. The present study is the first to explore the chest HRCT and PFTs features in EopSS-ILD (≥65 years) patients. The major findings of this study include the following: “lung- onset” subgroup in EopSS had a higher incidence of UIP, while NSIP/OP was more common in the AopSS patients. Although there was no significant difference in ventilation dysfunction between these 2 groups, small airway disease was more common in the EopSS patients, while restrictive ventilatory dysfunction was more common in the AopSS patients. Interestingly, PFTs showed a lower FEV1/FVC (%) and a higher RV/TLC (%) in the EopSS patients, compared with the AopSS patients. However, most of the patients did not meet the standards of obstructive ventilatory dysfunction. The level of serum CA19-9 in the EopSS patients was higher, compared with the AopSS patients.

Although pSS can occur at any age, middle-aged women are predominantly affected. Studies have shown that age may modify the phenotypes of autoimmune diseases, such as SLE and RA.^[6,7]^ There are few reports about the clinical features of pSS-ILD associated with the age of disease onset. Our previous studies had shown that the average age of pSS-ILD patients was (61.61 ± 10.99) years, and >60% of patients were ≥60 years.^[21]^ Garcia-Carrasco et al reported that the incidence of ILD was 13% in the elderly (≥70 years) and 8% in adults (<70 years) among 223 pSS patients based on the age of onset.^[22]^ Recently, Lee et al retrospectively analyzed 221 pSS patients and showed that the prevalence of ILD in pSS patients was 19.5% and ~51.2% in the elder patients (aged ≥65 years) and 15% in the adult patients (aged <65 years).^[10]^ Studies have suggested that the aged lung microenvironment leads to a reduction in tissue-resident alveolar macrophages, which play an important role in resolving inflammation after injury.^[23]^ In addition, tolerance of trachea mucous membrane to external antigen stimulation weakens during aging.^[24]^ Therefore, age might be an independent risk factor for ILD and more attention is needed for lung disorders in elderly pSS patients.

The high incidence of ILD in elderly patients can be due to the longer course of the disease or there may be a specific pathogenesis that needs further study. Lee et al confirmed that EopSS patients had a significantly shorter disease duration but a higher prevalence of pSS-related ILD, suggesting the association of older age of onset (not long-standing pSS) with the prevalence of ILD.^[10]^ In this study, we explored the characteristics of chest HRCT patterns and PFTs in the pSS-ILD patients according to the age of pSS-onset.

All pSS-ILD patients in this study had lung HRCT and PFTs that were used to evaluate the morphologic changes and dysfunction of the lung. Our study showed that multiple patterns of HRCT finding (OP/NSIP/UIP) were the most common feature, followed by UIP, NSIP, unclassified, OP, and LIP. The coexistence of diverse abnormalities suggests that different histopathological patterns can coexist in one patient, indicating the existence of nonuniformity and overlap of abnormalities.^[15]^ Gao et al reported that NSIP was the main pattern in patients with “lung-onset,” followed by the UIP-late pattern.^[25]^ Few reports have identified the HRCT features associated with the onset age in pSS-ILD patient. Lee KA et al analyzed the data from a total of 45 pSS-ILD patients and reported that there was no difference in the HRCT patterns (NSIP, UIP, OP, and LIP) between the elderly-onset and adult-onset groups.^[10]^ Their findings might be due to the limited number of cases.

In the present study, 102 pSS-ILD patients were grouped by age and stratified by initial symptoms. UIP was more common in the EopSS patients with “lung-onset,” compared with the AopSS patients. The results suggested that UIP was likely the main manifestation of HRCT, rather than NSIP/OP in the EopSS patients with the “lung -onset.” Our research further showed that ILD was more likely to develop chronic fibrosis patterns in EopSS patients.^[26]^ pSS is a proliferative disease of B lymphocytes. Studies on aging have shown that aging results in a significant decrease in the function of B lymphocytes and their subsets. Moreover, aging may decrease antibody response to exogenous antigens and vaccines and increase susceptibility to infection.^[27]^ Although ILD is relatively common in EopSS patients, inflammation reaction is weaker, and the process is persistent and chronic. The AopSS patients showed a significantly higher frequency of NSIP (single) and multiple with NSIP patterns. Patterns of OP (single) and multiple OP patterns were more common in the AopSS patients. These findings suggested that inflammation was more prominent in the AopSS patients. This phenomenon can be explained by the immune status of the lung in young patients. The lung is rich in blood vessels, and the immune response to exogenous antigen stimulation is stronger in young patients, leading to a more prominent inflammation. Several studies indicate that parotid enlargement, thyroiditis, vasculitis, and renal involvement are more common in young pSS patients.^[28]^ Therefore, the regulation mechanisms of aging in autoimmune rheumatic disease are very complex and the differences in underlying pathogenesis may lead to differences in disease phenotypes in various age groups.

In pSS-ILD patients, PFTs may detect a restrictive ventilatory dysfunction, characterized by reduced FVC, DLCO, and increased FEV1. In the early stage, PFTs impairment may be characterized by a reduced DLCO together with a preserved FVC. DLCO is highly sensitive in predicting the presence of ILD, whereas FVC may be more useful for assessing disease extent. The reduction in DLCO and the stability of FVC may be due to inflammation-induced thickening of the alveolar membrane.^[29,30]^

Lee et al evaluated FVC and DLCO, and their results demonstrated that patients (≥65 years) had a median FVC of 85% predicted and median DLCO of 69% predicted, while patients (<65 years) showed a median FVC of 78% predicted and median DLCO of 62% predicted.^[10]^ The present study demonstrated that the EopSS patients had (96.6 ± 26.7)% predicted and (72.6 ± 25)% predicted, respectively, while the AopSS group had (81.0 ± 21.2)% and (65.8 ± 24.8)%, respectively. Our results are similar to those of Lee et al, indicating that reduced DLCO and FVC are more common in the AopSS group. Therefore, the damage to diffusion capacity and restrictive ventilatory dysfunction are more prominent in the AopSS group, compared with the EopSS group.

HRCT data indicated that TLC, FVC, and VC significantly decreased in OP and multiple patterns. Impairment of lung function results in significant restrictive ventilatory dysfunction. OP was more common in the AopSS patients, which might explain the decreased FVC and VC. Furthermore, inflammation leads to the thickening of the alveolar membrane which results in reduced DLCO in AopSS patients. We further analyzed PFTs in the “sicca-onset” and “lung-onset” subgroups based on initial symptoms. TLC, FVC, and VC significantly decreased in the “lung- onset” group in AopSS patients, leading to restrictive ventilatory dysfunction. Whereas, FVC decreased slightly in the EopSS group, accompanied by obstructive ventilatory dysfunction with lower FEV1/FVC (%) and higher RV/TLC (%). Although there was no significant difference between these 2 subgroups, small airway disease was more common in the EopSS patients compared with the AopSS patients. This airway damage may be due to the older age or illness itself. Previous studies also indicated that pSS patients had various pulmonary presentations, including ILD (complex pathological patterns), airway disease, and pleural involvement. Therefore, physicians need to give a full evaluation of the lung functions of the EopSS patients.

Goules et al had shown that pSS patients with late-onset had a high incidence of dry eyes, peripheral neuropathy, and involvement of the central nervous system, while arthralgia/myalgia and lymphadenopathy were less common.^[31]^ Botsios et al reported that 71.4% of elderly-onset patients had dry mouth and 76.1% had dry eye. The most common extra glandular manifestation was arthritis (66.7%), followed by Raynaud phenomenon (23.8%) and purpura (4.2%).^[8]^ The frequency of respiratory symptoms as the main complaint at initial diagnosis was higher in elderly onset (28.5%) than in adult-onset (16%).^[10]^ These results indicate that symptoms associated with exocrine gland involvement and systemic nonspecific symptoms are more prominent in elderly patients. In contrast, Lee et al and Botsios et al found that sicca was not significantly different between the EopSS and AopSS patients.^[8,10]^ Our results indicated that sicca, weight loss, dry cough, and fatigue were higher in the EopSS patients, but the differences were not statistically significant. Sicca is influenced by many factors, such as mucosal tolerance disorder with aging. Therefore, elderly patients are prone to refractory dry mouth, dry eyes, irritant cough, and other symptoms.^[24]^ In addition, we found that arthritis, Raynaud, and parotid enlargement were more common in the AopSS patients, which was consistent with other studies.^[8,10]^ These clinical features are similar to lung characteristics (NSIP/OP), suggesting inflammation is prominent in AopSS patients.

Studies show that pSS patients with late disease onset have lower levels of autoantibodies such as RF, ANA, anti-Ro/SSA, or anti-La/SSB antibodies in comparison to younger pSS patients.^[8,10,28,32]^ Our results demonstrated that the level of inflammatory biomarkers (ESR) was higher, and the level of C4 was lower in the EopSS patients. The level of IgG, CRP, and a positive test of autoantibodies (ANA, anti-Ro/SSA, anti-La/SSB, and RF) were not significantly different between these 2 groups. The higher inflammatory biomarkers could be related to other complications in EopSS patients.

Serum tumor markers reflect proliferation and secretory activity of alveolar epithelial cells. Recent studies have confirmed that the level of serum tumor markers is dramatically higher in connective tissue disease patients with ILD, which has been used as a predictor for ILD onset and deterioration.^[33,34]^ Studies have shown that the serum levels of CA125, CEA, and CA153 were elevated in the pSS-ILD.^[35]^ CA19-9 can be used as a biomarker for lung disease severity in patients with end-stage ILD. The present study demonstrates that EopSS patients have significantly elevated serum levels of CA19-9. Moreover, the serum level of CA19-9 shows a strong association with age. The mechanisms leading to the high CA19-9 level in ILDs remain unclear. One study showed that regenerating epithelial cells in damaged lungs can release excessive CA19-9 and serum CA19-9 levels may reflect the worsening of ILD due to the extensive regeneration of epithelial cells.^[36]^ Further study is needed to validate the correlation between CA19-9 and end-stage lung disease in pSS-ILD.

The present study has several limitations. First, this is a retrospective study. Second, this is a single-center study, which may limit the generalizability of the results. Third, we only focus on the HRCT of the pSS-ILD patients, and the data on treatment and prognosis are not included. Further studies are needed with HRCT quantification to compare the severity of ILD between elderly-onset patients and adult-onset patients.

In conclusion, it is the first study that investigates the lung HRCT and PFTs features in EopSS-ILD patients. Our HRCT evaluation shows that UIP is the most common pattern in the “lung-onset” subgroup of EopSS patients. NSIP/OP is more common in the AopSS patients. The degree of damage in lung function is different in PFTs analyses. Although there is no significant difference in ventilation dysfunction between EopSS and AopSS groups, small airway disease is more common in the EopSS group, while the damage of diffusion capacity and restrictive ventilatory dysfunction are more prominent in the AopSS group. ILD is persistent and chronic in EopSS patients.

## Author contributions

**Conceptualization:** Yongfeng Zhang.

**Data curation:** Xin Dong, Man Li, Dong Wang.

**Formal analysis:** Xin Dong, Man Li.

**Investigation:** Xin Dong, Yanli Gao, Dong Wang.

**Methodology:** Dong Wang, Jifeng Li.

**Project administration:** Man Li, Jifeng Li, Yongfeng Zhang.

**Resources:** Jifeng Li.

**Software:** Xin Dong, Yanli Gao, Man Li, Dong Wang, Yongfeng Zhang.

**Validation:** Yongfeng Zhang.

**Writing – original draft:** Xin Dong, Yongfeng Zhang.

**Writing – review & editing:** Jifeng Li.
